# A *Symphytum officinale* Root Extract Exerts Anti-inflammatory Properties by Affecting Two Distinct Steps of NF-κB Signaling

**DOI:** 10.3389/fphar.2019.00289

**Published:** 2019-04-26

**Authors:** Jacqueline Seigner, Marc Junker-Samek, Alberto Plaza, Gilda D‘Urso, Milena Masullo, Sonia Piacente, Yvonne M. Holper-Schichl, Rainer de Martin

**Affiliations:** ^1^Department of Vascular Biology, Medical University of Vienna, Vienna, Austria; ^2^R&D, Procter & Gamble Health, Darmstadt, Germany; ^3^Dipartimento di Farmacia, Università degli Studi di Salerno, Salerno, Italy; ^4^Drehm Pharma GmbH, Vienna, Austria

**Keywords:** comfrey, *Symphytum officinale*, endothelial cells, inflammation, NF-κB, transcription, transactivation

## Abstract

*Symphytum officinale*, commonly known as comfrey, constitutes a traditional medicinal plant with a long-standing therapeutic history, and preparations thereof have been widely used for the treatment of painful muscle and joint complaints, wound and bone healing, and inflammation. Today, its topical use is based on its analgesic and anti-inflammatory effects, which have been substantiated by modern clinical trials. However, the molecular basis of its action remained elusive. Here, we show that a hydroalcoholic extract of comfrey root impairs the development of a pro-inflammatory scenario in primary human endothelial cells in a dose-dependent manner. The extract, and especially its mucilage-depleted fraction, impair the interleukin-1 (IL-1) induced expression of pro-inflammatory markers including E-selectin, VCAM1, ICAM1, and COX-2. Both preparations inhibit the activation of NF-κB, a transcription factor of central importance for the expression of these and other pro-inflammatory genes. Furthermore, our biochemical studies provide evidence that comfrey inhibits NF-κB signaling at two stages: it dampens not only the activation of IKK1/2 and the subsequent IκBα degradation, but also interferes with NF-κB p65 nucleo-cytoplasmatic shuttling and transactivation. These results provide a first mechanistic insight into the mode of action of a century-old popular herbal medicine.

## Introduction

*Symphytum officinale* (Boraginaceae), well known as comfrey, represents a plant with an impressive record of medicinal use. Native in Europe, it has been used for centuries for the treatment of a variety of painful muscle and joint complaints (Staiger, 2012; Frost et al., 2013). Phenolic compounds have been reported to exert anti-inflammatory effects both *in vitro* and *in vivo* (Chen et al., 2018). Several cellular mechanisms have been proposed in order to explain their mode of action, including the targeting of different intracellular signaling pathways triggered by NF-κB, AP-1, PPAR, Nrf2, and MAPKs (Chen et al., 2017; Tasneem et al., 2019). Today, the efficacy and safety of comfrey root extract ointment has been substantiated by several randomized clinical trials and non-interventional studies. Comfrey was proven effective for the treatment of, e.g., acute upper and lower back pain, gonarthrosis, or for patients with blunt injuries (Koll et al., 2004; Predel et al., 2005; Grube et al., 2007; Giannetti et al., 2010). Specified therein, its application is shown to significantly reduce pain and swelling, contribute to tissue regeneration, and result in a more rapid functional improvement. An assessment from the German Commission E resulted in the positive evaluationof comfrey root derived from *S. officinale* L. for the external use in bruises, strains, and sprains and led to the acknowledgment of its anti-inflammatory action (Kommission, 1990). In addition, the European Scientific Cooperative on Phytotherapy provides information about the use of comfrey for tendinitis syndrome, knee joint injuries, non-active gonarthrosis, insect bites, mastitis, fractures, and skin inflammation (ESCOP, 2009). To date, the therapeutic value of preparations from comfrey root is widely accepted, and they have been marketed in more than 10 countries.

The constituents of comfrey root include allantoin, mucilage polysaccharides, phenolic compounds including rosmarinic acid, chlorogenic acid, caffeic acid and derivatives, glycopeptides, and triterpene saponins (Staiger, 2012; Sowa et al., 2018). Comfrey also contains pyrrolizidine alkaloids such as 7-acetylintermedine, 7-acetyllycopsamine, intermedine, lycopsamine, and symphytine (Brauchli et al., 1982) that can cause liver toxicity (Ridker et al., 1985; Stickel and Seitz, 2000; Trifan et al., 2018), and, thereby, some medicinal products use pyrrolizidine alkaloid-depleted extracts as active ingredients.

Inflammation occurs in response to a wide variety of stimuli including microbial, physical (e.g., trauma) or chemical injury and it is essentially a beneficial reaction, since it represents the first response of the organism to “injury.” However, inflammation can turn into a chronic state that leads to the destruction of the involved body structures due to the generation of powerful biochemical mediators. One of the key events during the inflammatory episode is the activation of endothelial cells in blood vessels near the site of injury, which allows the chemoattraction, adhesion, and transmigration of leucocytes from the blood stream into the underlying affected tissue. To enable this, endothelial cells express a wide variety of pro-inflammatory genes including cell adhesion molecules (E-selectin, P-selectin, VCAM-1), cytokines and chemokines [interleukin (IL)-1, IL-6, IL-8], enzymes (COX-2, iNOS, SOD), and many others (Mayer et al., 2004). The transcription factor NF-κB has been widely accepted as a master regulator for the expression of these genes. NF-κB is activated through signaling pathways that originate at specific receptors (IL-1, TNF, LPS), proceed through receptor-specific adapter molecules and kinases (e.g., TAK1), and converge at the IκB kinase (IKK) complex that consists of the IKKs 1 and 2, and the regulatory subunit IKKγ/NEMO. When activated, the IKK complex phosphorylates IκBα/NFKBIA, an inhibitor of NF-κB that under non-stimulated conditions retains the transcription factor in the cytoplasm. In turn, phosphorylation is a signal for ubiquitin-dependent degradation of IκBα, thereby liberating NF-κB from its inhibitor and enabling its translocation to the nucleus, where it directs the expression of the pro-inflammatory genes described above (for a review, see de Martin et al., 2000; Karin, 2006).

Despite several clinical studies demonstrating the efficacy of comfrey root extracts and knowledge of several of its secondary metabolites (Koll et al., 2004; Predel et al., 2005; Grube et al., 2007), there is only insufficient insight into its mode of action on the molecular level. Therefore, we investigated the anti-inflammatory properties of a hydroalcoholic comfrey root extract in an *in vitro* model of inflammation. Our results indicate that the extract, and its mucilage-free organic fraction, can interfere with the activation of NF-κB and hence with key pro-inflammatory genes which are regulated by this transcription factor.

## Materials and Methods

### *S. officinale* Extract

A hydroalcoholic (20% ethanol w/w) liquid extract of comfrey roots (DER 1:2), termed comfrey-RE, was obtained from Merck KGaA & Co. Werk Spittal, Austria. To prepare a mucilage-depleted fraction, termed comfrey-OP, the ethanol content of the liquid extract was evaporated and the aqueous phase was partitioned with ethylacetate (1:1). The organic phase was dried in a SpeedVac (SavanT), dissolved in 100% ethanol and further diluted with cell culture medium yielding a stock concentration of 10 mg/ml with a final ethanol concentration of 2%.

For characterization of the extract and its main compounds, the ethylacetate fraction was analyzed by HPLC-UV. The HPLC profile showed peaks corresponding to the main compounds which were collected and structurally elucidated by NMR spectroscopy. In this way, allantoin, protocatechuic acid, *p*-hydroxybenzoic acid, caffeic acid, rosmarinic acid, and globoidnan A were identified. Whereas the first five were previously isolated in comfrey roots (Grabias and Swiatek, 1998; Trifan et al., 2018), this is the first report of globoidnan A in *S. officinale*. In order to determine their amounts, a quantitative determination by LC-MS was carried out (Supplementary Figure S1). A detailed description of the procedure is given in the Supplementary Material.

### Cell Culture

Primary human venous endothelial cells were isolated from umbilical cords as described previously (Hoeth et al., 2012) and maintained in M199 medium (Lonza) supplemented with 20% FCS (Sigma), 2 mM L-glutamine (Sigma), penicillin (100 units/ml), streptomycin (100 mg/ml), 5 units/ml heparin, and 25 mg/ml ECGS (Promocell), and were used up to passage 5.

### Antibodies and Reagents

Antibodies were obtained from the following suppliers and used at the respective dilutions: IκBα (Cell Signaling, #9241; 1:1000), Phospho-IκBα (Cell Signaling, #2859; 1:1000), IKK2 (Cell Signaling, #8943; 1:1000), Phospho-IKK1/2 (Cell Signaling, #2694; 1:1000), β-actin (SantaCruz, #sc-1616; 1:2000), NF-κB p65 (Santa Cruz #sc-372, 1:500), E-selectin (R&D Systems, Clone BBIG-E4; 1:1000), Goat anti-Mouse IgG (HL) Cross-Adsorbed Secondary Antibody, HRP (Invitrogen; 1:5000), Donkey anti-Rabbit IgG HRP-linked whole AB (Sigma; 1:5000), Goat anti-Rabbit IgG (HL) highly cross-adsorbed secondary antibody, Alexa Fluor 488 (Invitrogen; 1:1000).

The TAK1 inhibitor (5Z)-7-oxozeaenol: (8-(5-chloro-2-(4-methylpiperazin-1-yl)isonicotinamido)-1-(4-fluorophenyl)-4,5-dihydro-1H-benzo[g)indazole-3-carboxamide and the IKK2 inhibitor PHA-408 were obtained from Sigma. Recombinant human IL-1β was from R&D Systems.

### Cytotoxicity Assays

For determination of cytotoxicity, human umbilical vein endothelial cells (HUVECs) were incubated for 6 h with different concentrations of extract and assayed using the CellTox Green Cytotoxicity Assay (Promega, #G8741) according to the manufacturer’s recommendations.

### Real-Time PCR

Total RNA was isolated using the PeqGold Total RNA Isolation Kit (VWR International) according to the manufacturer’s instructions; 1 μg RNA was reverse transcribed using random hexamers (Fermentas) and murine leukemia virus reverse transcriptase (Thermo Scientific Fisher). Primers were designed using the software “Primer3,” and sequences are given in Supplementary Table S1. Real-time PCR was done with the SsoAdvanced Universal SYBR Green Supermix (BioRad) using the StepOnePlus instrument (Applied Biosystems), and relative mRNA expression normalized to GAPDH. Fold changes in mRNA expression were calculated according to the 2^-ΔΔCT^ method. Results are presented as mean fold induction of averaged Ct values of triplicates.

### Cell ELISA

Postconfluent HUVECs in 96-well plates were treated for 30 min with comfrey extracts and then stimulated with IL-1β (5 ng/ml) in the same medium. After 2 h, cells were fixed with 4% paraformaldehyde for 15 min following a blocking with 2.5% bovine serum albumin (BSA) in Tris-Buffered Saline-1% Tween (TBS-T) for 1 h. Afterward, cells were incubated overnight at 4°C with mouse-anti-E-selectin antibody (R&D Systems) diluted 1:500 in 1% BSA/TBS-T. Following washing with TBS-T, goat-anti-mouse horseradish-peroxidase (HRP) antibody (Sigma) diluted 1:10,000 in 1% BSA/TBS-T was added for 1 h. HRP activity was assessed using tetramethylbenzidine (Sigma) as substrate. The reaction was stopped adding 2 M H_2_SO_4_ and absorbance measured at OD_450nm_. E-selectin levels were normalized to crystal violet staining.

### Western Blotting

Western blotting was done as described previously (Basilio et al., 2013). Briefly, total cellular protein was extracted from HUVECs by lysis with 2x Laemmli buffer, proteins separated by 10% SDS PAGE and transferred to nitrocellulose membranes (Amersham Biosciences) by semidry blotting. Membranes were blocked with TBS-T containing 5% non-fat dry milk for 1 h at room temperature and incubated with the primary antibody overnight at 4°C, followed by horseradish peroxidase-conjugated secondary antibodies and chemiluminescence detection (WesternBright Chemilumineszenz Substrat Sirius; Biozym Scientific GmbH).

### Reporter Gene Assays

Human umbilical vein endothelial cells were grown in 12-well plates and transfected using the jetPEI-HUVEC DNA transfection reagent (PolyPlus) using a total amount of 2 μg of DNA with 2 μg jetPEI in 100 μl 150 mM NaCl, which was added to 400 μl serum-free medium for 4 h. The following reporter and expression plasmids were used: pNL3.2.NF-κB-RE (Promega), SELE-luc [1.3 kb/MAM(ex)neo-luc], pmaxGFP (Amaxa). Expression levels of NanoLuc luciferase were analyzed using the NanoGlo Luciferase Assay (Promega) according to the manufacturer’s protocol. Firefly luciferase-based reporter gene assays were performed as described (de Wet et al., 1987). Experiments were performed in triplicates, and luciferase values normalized to co-transfected EGFP levels, and are depicted as mean fold induction.

For the transactivation assay, HUVECs were grown as above and transfected by electroporation using a BioRad Gene Pulser with the settings 200 V/960 μF; 5 × 10^6^ cells were electroporated in 400 μl RPMI medium in 0.4 cm cuvettes with a total of 10 μg plasmids. The reporter plasmid gal4-luc and the vectors for expression of the p65 transactivation domain (TAD) fused to the gal4 DNA binding domain (DBD; gal4/p65-TAD) and empty gal4 control (gal4/-), were kindly provided by M. L. Schmitz (Schmitz and Baeuerle, 1991).

### Cyclooxygenase Assays

For the determination of total COX activity in HUVEC, the COX Activity Assay Kit (Cayman Chemical, #760151) was used according to the manufacturer’s recommendations. Briefly, HUVECs were cultivated in 10 cm dishes and incubated with comfrey-RE or -OP for 30 min followed by IL-1β stimulation for 1.5 h. Afterward, cells were scraped into cold PBS and centrifuged at 2000 ×*g* for 10 min. Cell pellets were resuspended in 200 μl cold PBS, sonicated, and centrifuged at 10,000 ×*g* for 15 min. The supernatant was collected and used for analysis. For assaying the effect of comfrey extract on COX-1 enzymatic activity, recombinant COX-1 from the same assay was used.

### Immunofluorescence Microscopy

Postconfluent HUVECs cultured on fibronectin-coated glass coverslips were treated with comfrey-OP or the TAK inhibitor for 30 min prior to stimulation with IL-1β. At the indicated time points, cells were fixed for 15 min with 4% paraformaldehyde (Sigma), permeabilized for 30 min with 0.1% Triton X-100 (Sigma), washed with PBS, and finally blocked for 1 h with 3% BSA-TBS-T. For immunostaining, rabbit polyclonal anti-p65 antibody (Santa Cruz) was used (1:500) with a secondary antibody, Alexa-Fluor 488-conjugated goat anti-rabbit IgG (Invitrogen) at 1:1000. Cells were counterstained for 15 min with Alexa Fluor 568 phalloidin (1:1000) and 5 min with 4′,6-Diamidino-2-phenylindole (DAPI, Life Technologies; 1:10,000). Samples were examined with an Olympus IX71 microscope with a 20x/0.75 UPlanSApo objective. Images were processed with Image J software.

### Statistical Significance Calculations

Differences between samples were analyzed using paired Student’s *t*-test. Two-tailed probability values of <0.05 and <0.01 were considered significant and highly significant, respectively. *p*-values are given in the figure legends.

## Results

### *S. officinale* Extract Impairs Pro-inflammatory Gene Expression

We tested preparations of comfrey root extract (a hydroalcoholic liquid root extract, termed comfrey-RE and its mucilage-depleted fraction, termed comfrey-OP; see section “Materials and Methods”) for its potential anti-inflammatory activity using IL-1 induced expression of E-selectin and other pro-inflammatory genes in primary HUVECs. IL-1 is a well-described pro-inflammatory mediator that plays a central role in the regulation of immune and inflammatory responses not only in response to infections but also to sterile insults such as trauma and blunt injuries.

**FIGURE 1:**
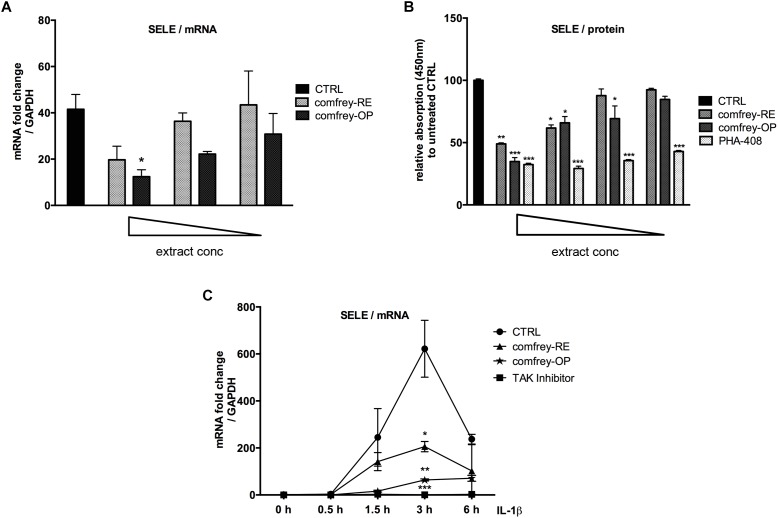
*S. officinale* root extract preparations affect E-selectin expression in a dose- and time-dependent manner. **(A)** Real-time PCR of IL-1β (5 ng/ml, 90 min) stimulated HUVEC which were either left untreated (CTRL, black bar) or pretreated (30 min) with either 166, 125, 100 μg/ml of comfrey-RE (gray dotted bars) or with 20, 10, 5 μg/ml of comfrey-OP (black dotted bars). Relative mRNA levels of E-selectin were normalized to GAPDH and expression levels are depicted as mean fold change compared to non-stimulated cells. **(B)** Cell ELISA of IL-1β (5 ng/ml, 90 min) stimulated HUVECs which were either left untreated (CTRL, black bar) or pretreated (30 min) with different doses (either 160, 130, 80, 60 μg/ml) of comfrey-RE (gray bars), comfrey-OP (20, 12, 10, 6 μg/ml; dark gray bars) or the inhibitor PHA-408 (20, 10, 5, 2.5 μM; light gray bars). E-selectin protein levels were analyzed after 2 h. **(C)** Real-time PCR of IL-1β induced (5 ng/ml; time points of stimulation as indicated) HUVEC either left untreated (CTRL, circles) or pretreated (30 min) with comfrey-RE (200 μg/ml; squares), comfrey-OP (20 μg/ml; asterisks) or the TAK1 inhibitor (5Z)-7-oxozeaenol (used as positive control at 5 μM). Relative mRNA levels of E-selectin were normalized to GAPDH and values are depicted as mean fold change compared to non-stimulated cells. Error bars represent mean ± SD (*n* = 3). ^∗^*p* < 0.05; ^∗∗^*p* < 0.01; ^∗∗∗^*p* < 0.001.

First, the non-toxic concentration range of both preparations was determined using the CellTox Green assay and found starting at a final conc. of 200 and 20 μg/ml for comfrey-RE and -OP, respectively (6 h incubation time; see Supplementary Figures S2A,B). Subsequently, dose-response and time course experiments were performed. As shown in Figure 1A, the IL-1 induced E-selectin mRNA levels were inhibited by comfrey-RE by approximately 50% at the starting concentration of 166 μg/ml, and by comfrey-OP by 70% at 20 μg/ml, indicating a more than 10-fold higher activity of the ethylacetate fraction. These results were also reflected at the protein level, where both comfrey-RE and -OP inhibited E-selectin protein expression at similar concentrations, as determined by cell ELISA (Figure 1B). Notably, pre-incubation with the extract for at least 30 min was necessary, as no inhibition was observed when applying it together with the stimulus (Supplementary Figure S2C).

**FIGURE 2:**
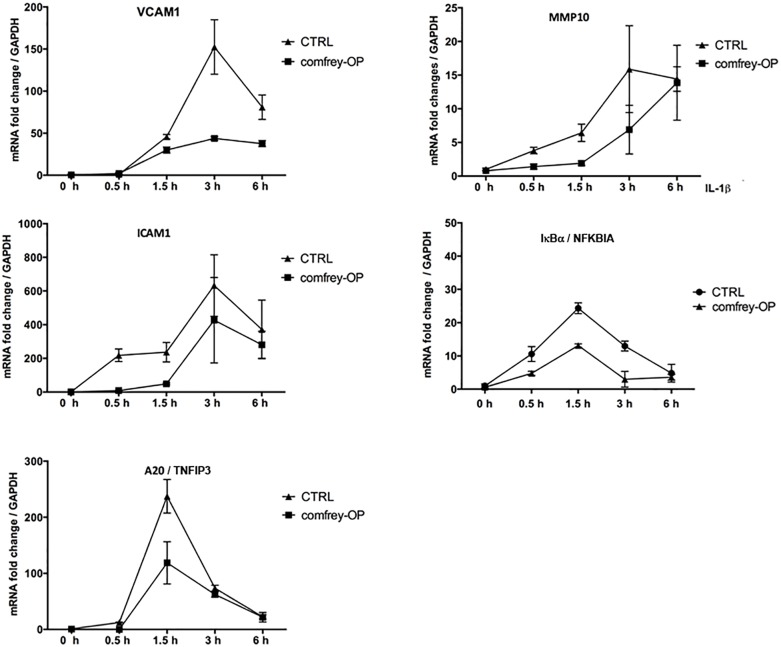
Comfrey-OP dampens the expression of various NF-κB-dependent genes. Real-time PCR of IL-1β induced (5 ng/ml; time points of stimulation as indicated) HUVEC that were either left untreated (CTRL, squares) or pretreated (30 min) with comfrey-OP (20 μg/ml; boxes). Relative mRNA levels of ICAM1 (intercellular adhesion molecule 1), A20 (TNFIP3; tumor necrosis factor alpha-induced protein 3), VCAM1 (vascular cell adhesion molecule 1), MMP10 (matrix metalloproteinase 10), and IκBα (NFKBIA; inhibitor of NF-κB, alpha) were normalized to GAPDH. Expression levels are depicted as mean fold change compared to non-stimulated cells. Error bars represent mean ± SD (*n* = 3).

To further determine possible effects of comfrey on the kinetics of E-selectin expression, we stimulated HUVEC with IL-1 for different times up to 6 hours in the presence of comfrey-RE and -OP. As shown in Figure 1C, E-selectin mRNA levels increased in response to IL-1 within 30 min (not visible due to scale), peaked at 3 h, and then declined. Both preparations of the extract diminished E-selectin expression at all time points, and confirmed the higher potency of comfrey-OP. Since IL-1 induces a plethora of genes in HUVEC with very different kinetics ranging from immediate-early to late-phase (Mayer et al., 2004), we tested others which are relevant in the context of vascular inflammation using comfrey-OP. As seen in Figure 2, in addition to E-selectin, the cell adhesion molecules VCAM-1 and ICAM-1 and the matrix metalloproteinase MMP10 were affected. Moreover, also the expression of IκBα (NFKBIA), an inhibitor of the transcription factor NF-κB, and of A20 (TNFAIP3), an anti-apoptotic gene, was diminished. This indicated that comfrey root extract has broader anti-inflammatory properties in IL-1 stimulated HUVEC.

### Inhibition of Cyclooxygenases by *S. officinale* Extract

**FIGURE 3:**
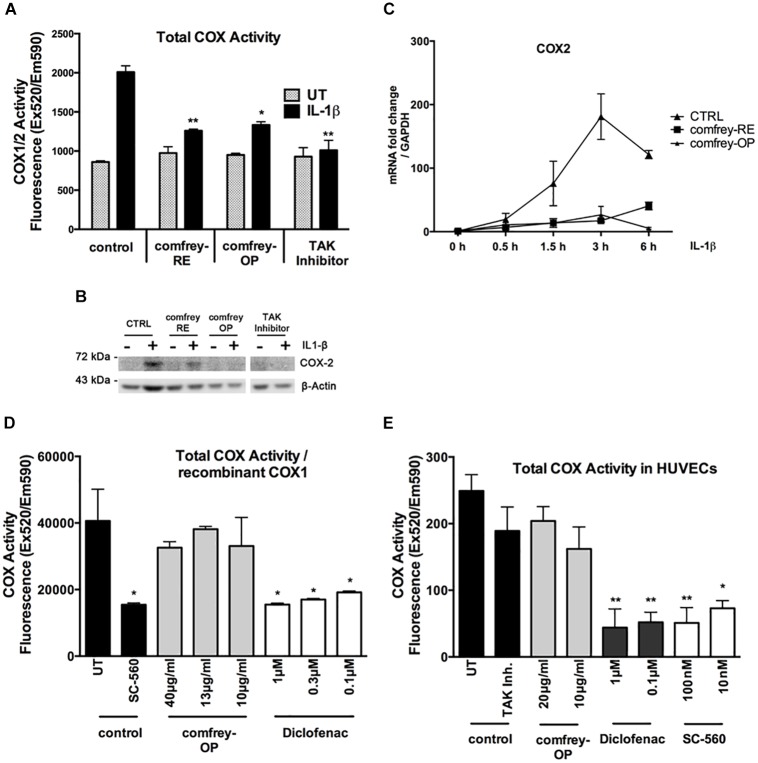
Comfrey-RE and -OP selectively block COX-2 but not COX-1. **(A)** Analysis of total COX-enzymatic activity (COX1/2) comparing unstimulated (UT, gray bars) and IL-1β (5 ng/ml, 90 min) stimulated HUVEC (black bars) which were either left untreated (control) or pretreated (30 min) with comfrey-RE (200 μg/ml), comfrey-OP (20 μg/ml), or TAK inhibitor (5 μM). Total COX enzymatic activity is depicted compared to non-stimulated, untreated cells. Error bars represent mean ± SD (*n* = 2). ^∗^*p* < 0.05; ^∗∗^*p* < 0.01. Note that both comfrey preparations diminish the IL-1β induced COX-2 activity (black bars), but not the constitutive COX-1 activity (gray bars). **(B)** Western blot analysis of samples used in **A** showing that COX-2 expression can only be found in IL-1β induced samples. Treatment of cells with comfrey preparations (RE and OP) reduces COX-2 protein levels. β-actin is shown as loading control. **(C)** Real-time PCR of IL-1β induced (5 ng/ml; time points of stimulation as indicated) HUVEC either left untreated (CTRL, squares) or pretreated (30 min) with comfrey-RE (200 μg/ml; boxes) and comfrey-OP (20 μg/ml; asterisks). Relative mRNA levels of COX-2 were normalized to GAPDH. Expression levels are depicted as mean fold change compared to non-stimulated cells. Error bars represent mean ± SD (*n* = 3). **(D)** Comfrey-OP does not affect COX-1 activity. *In vitro* analysis of recombinant COX-1 enzymatic activity comparing samples which were either left untreated (control, UT) or treated with the specific COX-1 inhibitor SC-560 (control, black bars) with samples incubated with different concentrations of comfrey-OP (gray bars) or diclofenac (white bars). Total COX enzymatic activity is depicted compared to untreated cells. Error bars represent mean ± SD (*n* = 3). ^∗^*p* < 0.05. **(E)** Analysis of total COX-enzymatic activity (COX1/2) comparing cells either left untreated (control, UT) or treated with TAK inhibitor (5 μM) (control, black bars) with cells incubated with different concentrations (as indicated) of comfrey-OP, diclofenac, or the COX-1 inhibitor SC-560. Total COX enzymatic activity is depicted compared to untreated cells. Error bars represent mean ± SD (*n* = 3). ^∗^*p* < 0.05; ^∗∗^*p* < 0.01.

Given the importance of cyclooxygenases in the inflammatory process, we have investigated whether comfrey extract can inhibit COX-1 and/or COX-2. First, HUVECs were stimulated with combinations of IL-1 and comfrey-RE or -OP, and COX enzymatic activity was determined using a fluorescence-based assay that measures both COX-1 and -2 (see section “Materials and Methods”). Comfrey-RE or -OP both significantly reduced the IL-1-induced, but not the constitutive COX activity (Figure 3A); since it is COX-2 which is induced by IL-1, whereas COX-1 is constitutively expressed, it can be concluded that comfrey selectively impacts COX-2 activity. Western analysis of the same samples using a COX-2 specific antibody confirmed that COX-2 protein levels were induced by IL-1 and diminished by comfrey-OP and -RE (Figure 3B). Besides, when reanalyzing the samples shown in Figure 2, COX-2 mRNA was lower in comfrey-OP treated cells as compared to control (Figure 3C). The potential selectivity of comfrey toward COX-2 was further tested by using recombinant COX-1. Whereas the non-specific inhibitor diclofenac readily inhibited the enzymatic activity of COX-1, comfrey-OP did not (Figure 3D). Finally, we treated non-stimulated HUVEC (which express only the constitutively present COX-1) with either the non-specific COX inhibitor diclofenac, the specific COX-1 inhibitor SC-560, or with comfrey-OP. This basal COX activity, which is attributed solely to COX-1, was inhibited by diclofenac and SC-560, but not by comfrey-OP (Figure 3E). Thus, we conclude that comfrey extract does not inhibit the enzymatic activity of both COX isoforms, but acts on COX-2 through prevention of its mRNA and protein synthesis, and therefore unlike diclofenac, acts indirectly through NF-κB inhibition, but specifically to inhibit COX-2.

### *S. officinale* Extract Affects NF-κB Signaling

**FIGURE 4:**
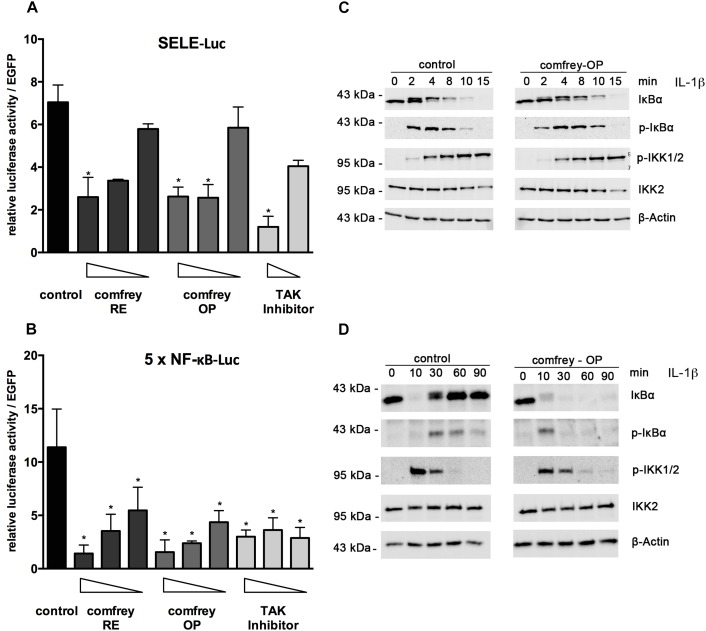
Comfrey inhibits NF-κB signaling. **(A)** Reporter gene assay in HUVEC transfected with an E-selectin promoter-reporter construct (SELE-Luc) and stimulated with IL-1 alone or in the presence of concentrations of comfrey-RE (200, 130, 100 μg/ml; dark bars), comfrey-OP (20, 13, 10 μg/ml; gray bars), or TAK inhibitor (5, 2.5 μM; light bars). Luciferase levels were assayed 16 h later and normalized to the fluorescence from a co-transfected EGFP plasmid used as transfection control. Values are shown as mean fold change in regard to non-stimulated cells. Error bars represent mean ± SD (*n* = 3). ^∗^*p* < 0.05. **(B)** Reporter gene analysis as described in **A**, except that an NF-κB-specific-reporter gene (5 × NF-κB-Luc) was used. **(C)** Western blot of IL-1β induced HUVEC (time points as indicated) either left untreated (control, left panels) or preincubated with comfrey-OP (right panels) for 30 min. Samples were analyzed for the presence of IκBα, phospho-IκBα (pIκBα), phospho-IKK1/2 (pIKK1/2), and total IKK2. β-actin represents the loading control. Note that the slower migrating band seen within the panel depicting IκBα represents phospho-IκBα. **(D)** Western blot as in **C**, except that the time course was extended to 90 min in order to capture the phase of IκBα re-synthesis.

To gain more mechanistic insight into the anti-inflammatory mode of action of comfrey, we investigated how the extract inhibits the expression of E-selectin. For assaying transcriptional regulation, we performed reporter gene assays using an E-selectin promoter-luciferase reporter. Treatment with comfrey-RE or -OP reduced the activity of the E-selectin promoter, suggesting an effect on transcriptional regulation (Figure 4A). Since NF-κB is a well-documented key regulator of E-selectin and of pro-inflammatory gene expression in EC in general, we extended our experiments by using an artificial NF-κB promoter containing a multimerized NF-κB binding site, a construct that can be used to specifically monitor NF-κB activity. Again, comfrey-RE or -OP reduced NF-κB activity (Figure 4B). To further dissect the pathway and identify the level of interference, we performed Western analysis of key signaling molecules. A main regulatory step of NF-κB regulation is the phosphorylation, ubiquitination, and proteolytic degradation of its inhibitor IκBα, thereby liberating the transcription factor and allowing its translocation to the nucleus. The phosphorylation and degradation steps, which take place within minutes after IL-1 stimulation, were found to be diminished and delayed in comfrey-OP treated HUVEC as determined by Western blotting using anti-IκBα and anti-phospho-IκBα antibodies (Figure 4C). Moreover, IκBα re-synthesis which starts around 30 min post stimulation and is as well NF-κB dependent, was suppressed (Figure 4D). Since IκBα is phosphorylated mainly by IKK2, which is part of the IKK complex consisting of IKK1, IKK2, and IKKγ/NEMO (Mercurio et al., 1997), we also investigated whether comfrey may inhibit the activation of this kinase. IKK2 activity can be assayed indirectly by probing for the phosphorylation of residues in the activation loop of the kinase. Our Western analysis indicated that phosphorylation of the IKKs (the antibody detects both IKK1 and -2) was attenuated or delayed, although the effect was weak (Figure 4C,D). From these experiments, we conclude that comfrey inhibits NF-κB by interfering with the activation pathway at least in part at the level of IκBα phosphorylation and possibly of IKK activation.

**FIGURE 5:**
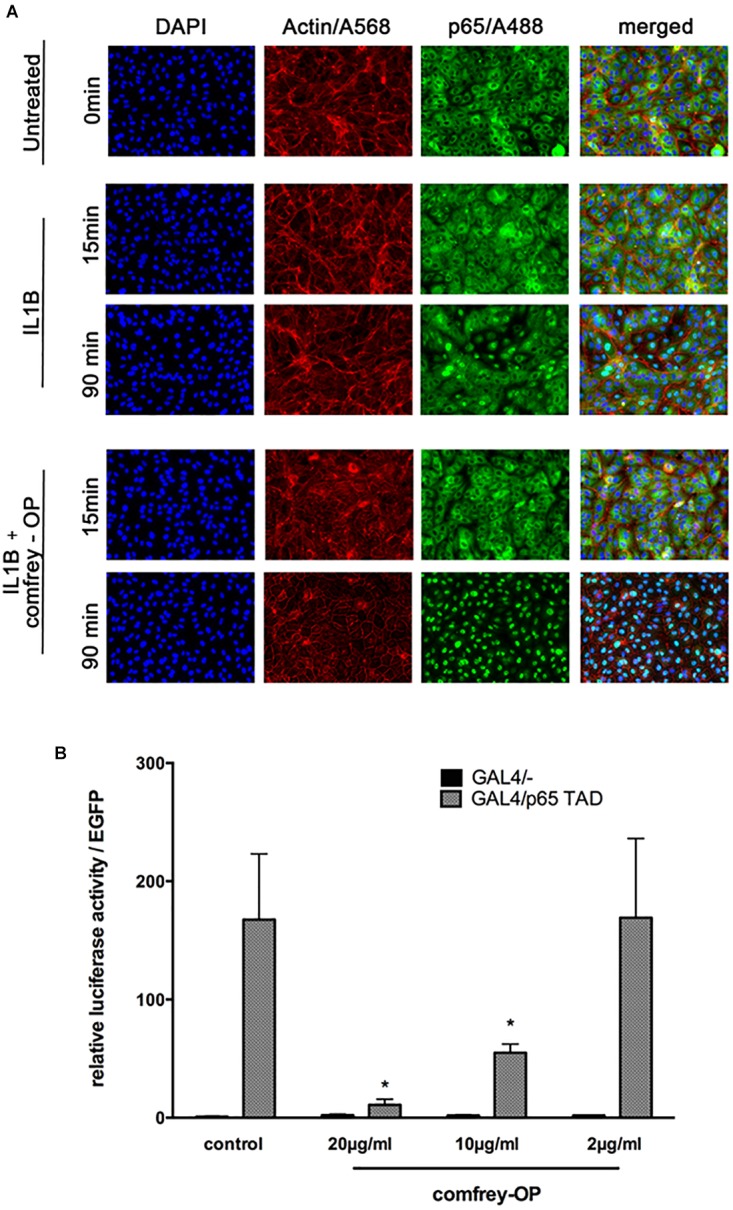
Comfrey interferes with NF-κB p65 nucleo-cytoplasmatic shuttling and with its transactivation. **(A)** Immunostaining of HUVECs that were stimulated with IL-1β alone or in combination with comfrey-OP (20 μg/ml) for 15 and 90 min as indicated. The p65 subunit of NF-κB is represented in green (A488), actin in red (A568), and intact nuclei in blue (DAPI); merged images are shown on the right. **(B)** Reporter gene assay in HUVEC transfected with a gal4 promoter-dependent luciferase reporter gene either in the presence of the Gal4/- control plasmid (black bars; does not activate reporter gene due to absent transactivation potential) or the activating plasmid, where the gal4 DNA binding domain is fused to the p65 transactivation domain (gal4-p65TAD, gray bars); 48 h post transfection cells were treated with comfrey-OP as indicated and analyzed for luciferase expression 16 h later. Values were normalized to co-transfected EGFP fluorescence, and are shown as mean fold induction in regard to non-stimulated cells. Error bars represent mean ± SD (*n* = 3). Significance levels as compared to gal4/p65 TAD are indicated (^∗^*p* < 0.05).

**FIGURE 6:**
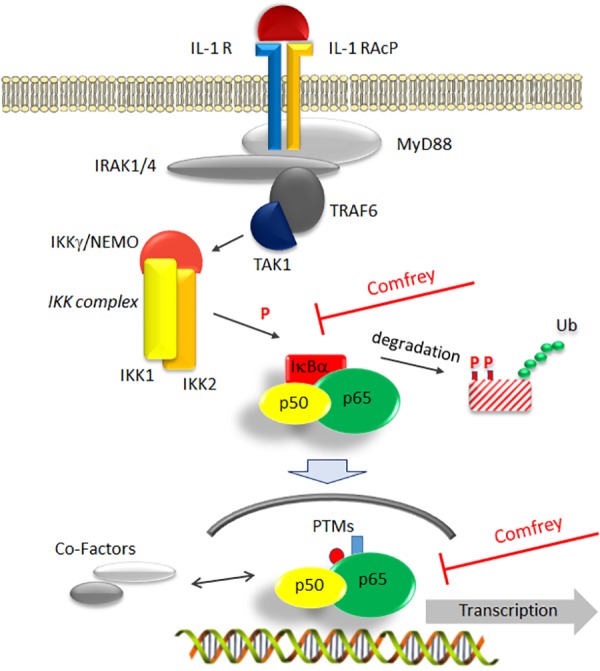
Model of comfrey’s mode of action by interference with the NF-κB signaling pathway. IL-1β binding to its receptor (IL-1 R) recruits an accessory protein (IL-1 RAcP) to activate a downstream signaling cascade involving MyD88, IRAK, and TRAF6. Of central importance is the activation of the IKK complex consisting of IKK-1, -2, and NEMO/IKKγ, which triggers the degradation of the main NF-κB inhibitor IκBα through phosphorylation (P) and ubiquitination (Ub). Liberation from IκBα enables NF-κB (depicted as a p50/p65 heterodimer) to translocate to the nucleus. Here, NF-κB can be modified further (post-translational modifications, PTMs) and interact with a variety of cofactors such as histone modifying proteins that modulate its transactivation potential. Based on our studies, the hydroalcoholic extract of comfrey root, as well as the mucilage-depleted fraction thereof, interfere at two points with this signaling cascade as indicated: first, IκBα phosphorylation and its subsequent degradation are delayed and second, transactivation of nuclear p65 becomes impaired. Together, this results in the alteration of pro-inflammatory gene expression, thus attenuating the further development of a pro-inflammatory scenario.

Moreover, to investigate if comfrey may act through additional mechanisms, we performed immunofluorescence staining for p65 (RelA) translocation into the nucleus after stimulation with IL-1. In HUVEC, IL-1 causes translocation of the transcription factor into the nucleus within 15 min, followed by export into the cytoplasm after ca. 90 min. However, in comfrey-treated cells, the transcription factor was retained in the nucleus at 90 min, suggesting that the extract interferes also with nuclear export (Figure 5A). A more detailed time course is depicted in Supplementary Figures S3A,B. Since this finding would predict an enhanced rather than a diminished activity of NF-κB, we reasoned that the transcription factor may reside in the nucleus in an inactive form. Since modifications in the TAD have been reported to influence NF-κB activity, we tested its transactivation potential using a gal4/p65TAD reporter system. The activity of this reporter was readily inhibited by comfrey (Figure 5B), suggesting that the tested hydroalcoholic comfrey root extract suppresses NF-κB on two levels of its signaling cascade: first, on the level of IKK/IκBα complex activation, and further on the level of p65 transactivation, thereby attenuating the expression of pro-inflammatory genes (Figure 6).

## Discussion

A common denominator of a broad spectrum of muscle and joint-related disorders is inflammation. Here, we utilized an *in vitro* model to assay the effect of a hydroalcoholic extract of comfrey root on one of the key steps in this process, namely, the IL-1 induced expression of E-selectin in endothelial cells (Smith, 1993). Two forms of IL-1, IL-1α and -β, bind to the same receptor(s) and evoke the same signaling pathway(s), however, are processed differently from their respective precursors, namely by ICE/caspase-1 and calpain, respectively. Whereas calpain is activated especially upon loss of plasma membrane integrity, which occurs during necrosis, ICE/caspase-1 is activated on the inflammasome, and thus IL-1β represents the main pro-inflammatory and best-characterized form in this context (Voronov et al., 2013; England et al., 2014). Induced E-selectin then mediates the first step of the adhesion of leukocytes to the endothelium, leading to their subsequent transmigration through the vessel wall and extravasation into the inflamed tissue. Comfrey extract reduced E-selectin expression on the mRNA and protein level; it also reduced the expression of several other genes involved in the inflammatory process including additional cell adhesion molecules involved in transmigration such as ICAM-1 and VCAM-1. This demonstrates that comfrey extract does exert anti-inflammatory properties *in vitro*, substantiating the beneficial effects observed in animal and clinical studies. Moreover, the ethylacetate fraction of the extract that is devoid of mucilage showed similar results to those of the whole hydroalcoholic extract.

The two cyclooxygenase isoforms COX-1 and -2 are key enzymes in arachidonic acid metabolism that lead to the synthesis of prostaglandins, which are in turn responsible for some of the symptoms of inflammation such as pain and vascular leakage. In our experiments, the inhibitory action of the tested comfrey preparations was COX-2 specific, with no effect on COX-1 enzymatic activity. Of note, no direct inhibitory effect on the enzymatic activity was observed, but expression of COX-2 itself was efficiently blocked.

To further elucidate the molecular mechanism(s) of the observed inhibitory effects, we investigated whether the NF-κB signaling pathway was affected. We reasoned that NF-κB might be involved since it controls the expression of E-selectin and many other pro-inflammatory genes including those that we tested. First, reporter gene assays using an NF-κB-dependent E-selectin promoter construct, as well as an NF-κB-specific reporter, revealed that NF-κB activity was inhibited by comfrey in IL-1 stimulated HUVECs. Based on these findings, we sought to further identify the point of interference with the signaling pathway. Activation of NF-κB is a multistep process: it involves the activation of the IKK complex by MAP kinases such as TAK1 and the serine phosphorylation of IκBα by mainly IKK2. The latter serves as a signal for its K48 ubiquitination and subsequent degradation by the proteasome, enabling the translocation of NF-κB to the nucleus. The activation pathway also necessitates modifications of the p65 NF-κB DNA binding and transactivation domains by phosphorylation and/or acetylation, as well as interaction with other factors, e.g., histone deacetylases. Over the years, inhibitors have been developed for targeting NF-κB, including those that we used as positive controls in our assays, e.g., the TAK1 inhibitor (5Z)-7-oxozeaenol (Ninomiya-Tsuji et al., 2003) or the IKK2-specific inhibitor PHA-408 (Sommers et al., 2009). Among them, IKK2 inhibitors are the most prominent, and our data suggest that comfrey acts in part on this level, since the activation of IKKs and the subsequent phosphorylation and degradation of IκBα was diminished (Figure 4C,D).

Notably, additional analysis suggested another level of NF-κB regulation by comfrey. Immunostaining for p65 confirmed the previously observed rapid (15–30 min after IL-1 stimulation) nuclear translocation and subsequent (at 90 min) export to the cytosol (Figure 5A). However, treatment of the cells with comfrey-OP partially suppressed this nuclear export, leading to accumulation of nuclear p65. This finding is in line with the diminished IκBα re-synthesis observed in Western blots (Figure 4D), since the NF-κB-dependent synthesis of its inhibitor constitutes a negative feedback loop, and also with the reduced IκBα mRNA levels as shown in Figure 2 (lower right panel). Importantly, re-synthesis of IκBα is necessary for the dissociation of NF-κB from the DNA binding sites and its subsequent nuclear export (Zabel and Baeuerle, 1990). However, nuclear NF-κB has traditionally been associated with active NF-κB, e.g., in synovial fibroblasts of patients with rheumatoid arthritis (Handel et al., 1995; Marok et al., 1996). In contrast, our results suggest that due to comfrey treatment, the transcription factor may reside in the nucleus in an inactive form. This is reminiscent of the situation in, e.g., LPS tolerant monocytic cells, where NF-κB accumulates in the nucleus, but is transcriptionally repressed and unable to direct the expression of IL-1 in these cells (Rodriguez et al., 1999). Indeed, regulation of NF-κB in the nucleus has been described on the level of DNA binding and of transactivation (Perkins, 2006). Therefore, we have assayed the transactivation potential of p65 using a reporter gene system where the p65 TAD is fused to a heterologous DBD (from gal4). Co-transfection with a gal4 responsive reporter gene allows the study of p65 transactivation without interference from DNA binding. Treatment of IL-1 stimulated HUVEC with comfrey suppressed p65 transactivation in a dose-dependent manner (Figure 5B), suggesting that comfrey interferes with NF-κB activation also on the level of p65 transactivation. Several studies have demonstrated that specific phosphorylation reactions that lead further to acetylation and other posttranslational modifications, can influence the p65 transactivation potential (Sakurai et al., 1999; Anrather et al., 2005; Perkins, 2006; Sen et al., 2012). Notably, among the sites studied in more detail S468 and S536 have been reported as targets of IKK2 (Schwabe and Sakurai, 2005). One might therefore hypothesize that inhibition of phosphorylation of one or both of these sites by comfrey could be responsible for NF-κB inactivation in the nucleus. However, also nitrosylation reactions that affect redox-sensitive residues could be a target since they are reportedly affected by plant extracts and could possibly relate to the described anti-oxidant activity of comfrey (Natarajan et al., 1996; Sowa et al., 2018). Further work will aim at a more detailed elucidation of these mechanisms.

Taken together, our results provide a first insight into the molecular mechanism(s) exerted by this traditional medical plant. Moreover, the identification of NF-κB inhibition by comfrey root extract through targeting the transactivation process of p65 represents a less common feature as compared to the common IKK inhibitors, and may represent a novel lead for therapeutic interference.

## Author Contributions

JS performed the experiments and analyzed the results. AP and MJ-S contributed scientifically to the design of the experiments and analyzed the data. GD‘U performed LC-MS and HPLC experiments and interpreted the data. MM performed NMR experiments and interpreted the data. SP contributed with the data interpretation and supervision of LC-MS and NMR experiments. YH-S designed the project. RdM and YH-S designed the experiments, analyzed the data, and wrote the manuscript.

## Conflict of Interest Statement

This work was in part financially supported by Merck Selbstmedikation GmbH. The funder was involved in the study design, analysis, and interpretation of the data. The funder had no role in the collection of the data. AP and MJ-S are employees of Merck Selbstmedikation GmbH. YH-S is an employee of Drehm Pharma GmbH and engaged as a consultant for Merck. The remaining authors declare that the research was conducted in the absence of any commercial or financial relationships that could be construed as a potential conflict of interest.

## Supplementary Material

FIGURE S1 Characterization of *S. officinale* root extract and quantification of its main compounds. **(A)** The ethylacetate fraction of comfrey roots was analyzed by HPLC-UV. The HPLC profile showed peaks corresponding to the main compounds which were collected and structurally elucidated by NMR spectroscopy. In this way, allantoin (1), protocatechuic acid (2), *p*-hydroxybenzoic acid (3), caffeic acid (4), rosmarinic acid (5), and globoidnan A (6) were identified. Noteworthy, compounds 1–5 were previously isolated in comfrey roots (Grabias and Swiatek, 1998; Trifan et al., 2018) while this is the first report of globoidnan A (6). **(B)** In order to determine the amount of the main compounds occurring in the ethylacetate fraction of comfrey roots, a quantitative determination by LC-MS was carried out. LC/ESI/triple quadrupole(QqQ)/MS using a very sensitive and selective mass tandem experiment such as Multiple Reaction Monitoring (MRM) is considered one of the most suitable techniques for quantification of metabolites (Masullo et al., 2016). The amount (per mg/100 g of dried extract) of each selected compound and the MRM transition selected for each compound is shown. Allantoin (1) in the MS/MS spectrum was characterized by the loss of 60 Da corresponding to the urea unit, originating an intense peak at *m/z* 97. For this reason, this transition was selected for MRM experiments. Protocatechuic acid (2), *p*-hydroxybenzoic acid (3), and caffeic acid (4) showed a very simple fragmentation pattern where the base peak was produced by the neutral loss of 44 Da corresponding to the carboxylic group. MS/MS fragmentation pattern of rosmarinic acid (5) originated a main fragment ion [(M-180-H_2_O)-H]^-^ at *m/z* 161, due to the loss of a caffeic acid unit and water molecule. Globoidnan A (6) was characterized in the MS/MS spectrum by an intense peak [(M-180)-H]^-^ at *m/z* 311 due to the neutral loss of a caffeic acid unit. The quantitative results highlight that compounds are present in concentration ranges of 0.42–20.02 mg/100 g. In particular, globoidnan A (6) and rosmarinic acid (5) exhibit the highest concentrations of 20.02 mg/100 g and 14.03 mg/100 g of dried extract. DP: declustering potential; EP: entrance potential; CE: collision energy, PI: product ion, DI: daughter ion. Experimental details are given in the Supplementary Text.

FIGURE S2 Toxicity of different concentrations (as indicated) of comfrey-RE **(A)** or comfrey-OP **(B)** was assayed in HUVEC using the CellTox Green assay. Incubation time was 6 h. **(C)** Different concentrations of comfrey extract were added to IL-1β stimulated (90 min) HUVEC either 30 min before or at the same time. E-selectin mRNA was quantified by real-time PCR.

FIGURE S3 Detailed time course of p65 cellular distribution and its alteration by Comfrey-OP. Same experiment as in Figure 5A except that additional time points are shown. **(A)** IL-1β stimulated HUVEC. **(B)** comfrey-OP plus IL-1β stimulation.

TABLE S1 Primers for real-time PCR.
