# Supplementary figures and images for: A Symphytum officinale Root Extract Exerts Anti-inflammatory Properties by Affecting Two Distinct Steps of NF-κB Signaling
Source: Front Pharmacol. 2019 Apr 26;10:289. doi: 10.3389/fphar.2019.00289 (PMC6498879; doi:10.3389/fphar.2019.00289)

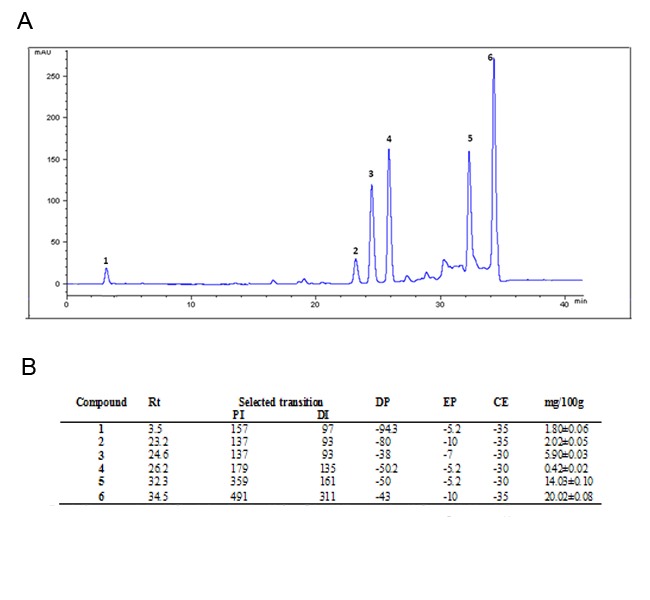

Supplement: FIGURE S1 — Characterization of S. officinale root extract and quantification of its main compounds. (A) The ethylacetate fraction of comfrey roots was analyzed by HPLC-UV. The HPLC profile showed peaks corresponding to the main compounds which were collected and structurally elucidated by NMR spectroscopy. In this way, allantoin (1), protocatechuic acid (2), p-hydroxybenzoic acid (3), caffeic acid (4), rosmarinic acid (5), and globoidnan A (6) were identified. Noteworthy, compounds 1–5 were previously isolated in comfrey roots (Grabias and Swiatek, 1998; Trifan et al., 2018) while this is the first report of globoidnan A (6). (B) In order to determine the amount of the main compounds occurring in the ethylacetate fraction of comfrey roots, a quantitative determination by LC-MS was carried out. LC/ESI/triple quadrupole(QqQ)/MS using a very sensitive and selective mass tandem experiment such as Multiple Reaction Monitoring (MRM) is considered one of the most suitable techniques for quantification of metabolites (Masullo et al., 2016). The amount (per mg/100 g of dried extract) of each selected compound and the MRM transition selected for each compound is shown. Allantoin (1) in the MS/MS spectrum was characterized by the loss of 60 Da corresponding to the urea unit, originating an intense peak at m/z 97. For this reason, this transition was selected for MRM experiments. Protocatechuic acid (2), p-hydroxybenzoic acid (3), and caffeic acid (4) showed a very simple fragmentation pattern where the base peak was produced by the neutral loss of 44 Da corresponding to the carboxylic group. MS/MS fragmentation pattern of rosmarinic acid (5) originated a main fragment ion [(M-180-H2O)-H]- at m/z 161, due to the loss of a caffeic acid unit and water molecule. Globoidnan A (6) was characterized in the MS/MS spectrum by an intense peak [(M-180)-H]- at m/z 311 due to the neutral loss of a caffeic acid unit. The quantitative results highlight that compounds are present in concentration ranges [file Image_1.TIF]

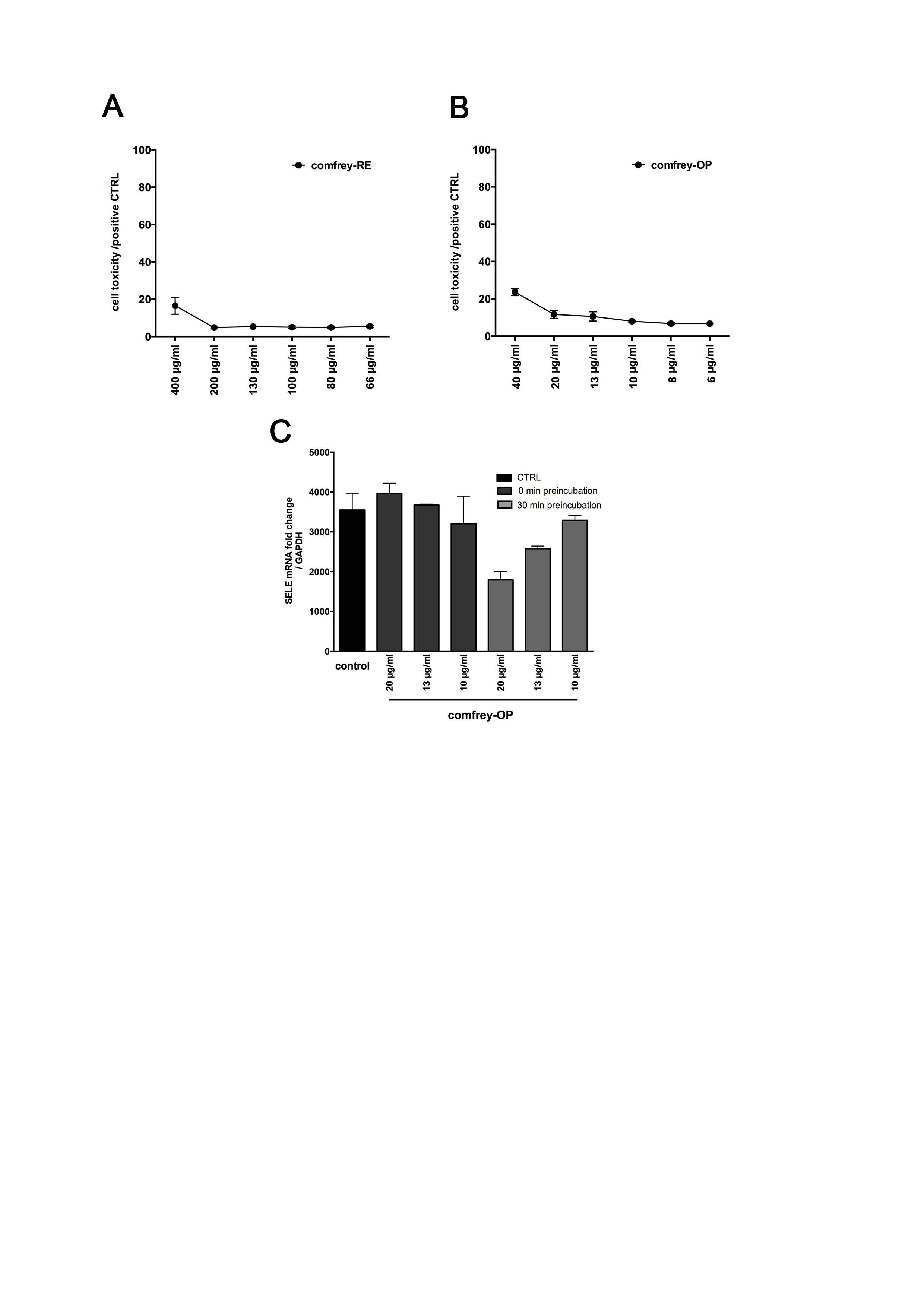

Supplement: FIGURE S2 — Toxicity of different concentrations (as indicated) of comfrey-RE (A) or comfrey-OP (B) was assayed in HUVEC using the CellTox Green assay. Incubation time was 6 h. (C) Different concentrations of comfrey extract were added to IL-1β stimulated (90 min) HUVEC either 30 min before or at the same time. E-selectin mRNA was quantified by real-time PCR. [file Image_2.TIF]

A

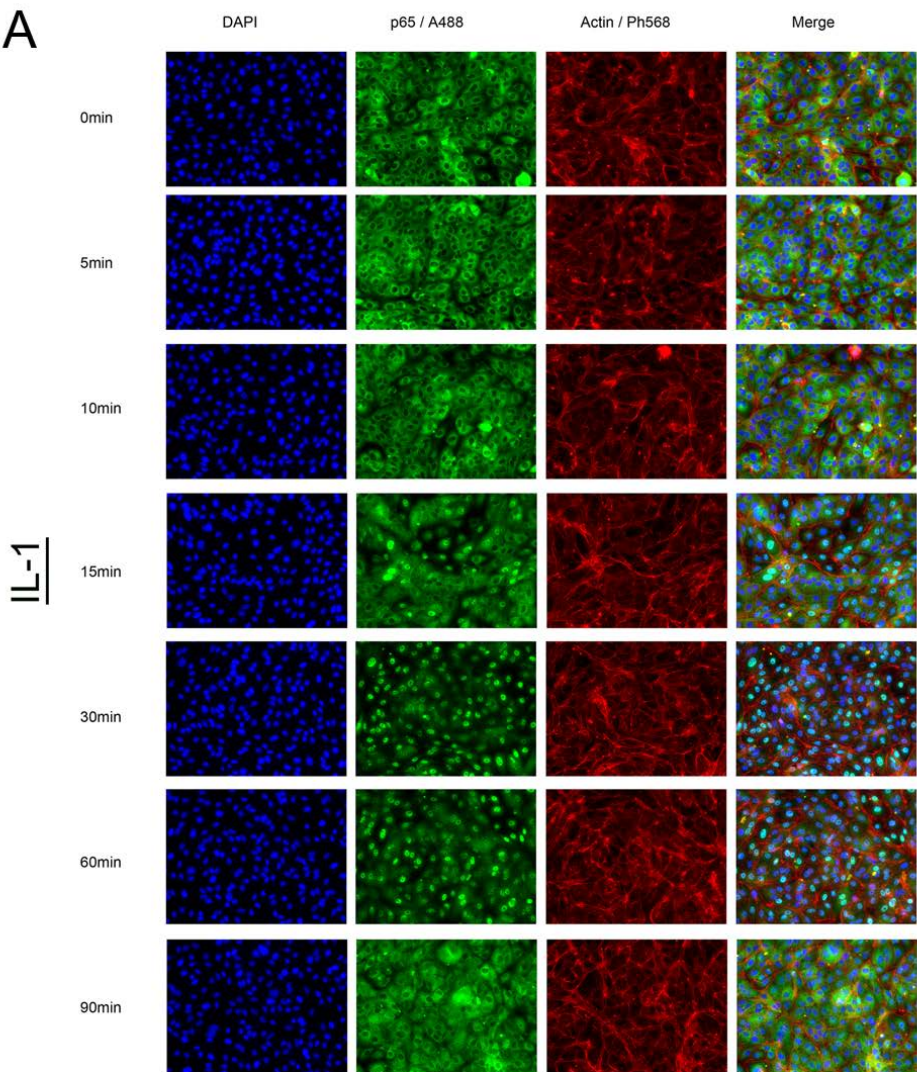

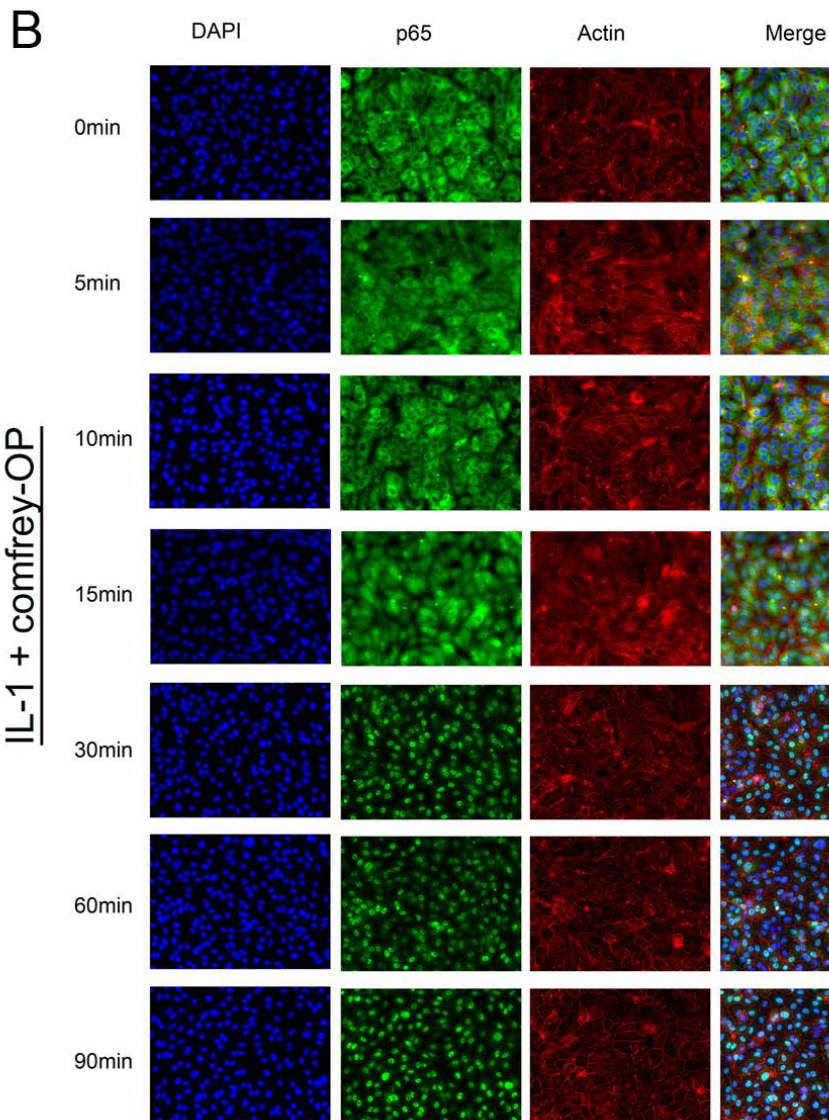

Supplement: FIGURE S3 — Detailed time course of p65 cellular distribution and its alteration by Comfrey-OP. Same experiment as in Figure 5A except that additional time points are shown. (A) IL-1β stimulated HUVEC. (B) comfrey-OP plus IL-1β stimulation. [file Image_3.pdf]
