# Supplementary material for: A Symphytum officinale Root Extract Exerts Anti-inflammatory Properties by Affecting Two Distinct Steps of NF-κB Signaling
Source: Front Pharmacol. 2019 Apr 26;10:289. doi: 10.3389/fphar.2019.00289 (PMC6498879; doi:10.3389/fphar.2019.00289)
Supplement: TABLE S1 — Primers for real-time PCR. [file Table_1.DOCX]

**SUPPLEMENTARY TABLE1 ǀ Primers for real-time PCR**

glyceraldehyde 3-phosphate dehydrogenase (GAPDH)

forward, 5´-AGAAGGCTGGGGCTCATTT-3´

reverse, 5´-CTAAGCAGTTGGTGGTGCAG-3´

E-selectin (SELE)

forward, 5´-CCTGTGAAGCTCCCACTGA-3´

reverse 5´- GGCTTTTGGTAGCTTCCATCT-3´

prostaglandin-endoperoxide Synthase 2 (PTGS-2/COX-2)

forward, 5´-CTGCTCAACACCGGAATTTT-3´

reverse, 5´-GAGAAGGCTTCCCAGCTTTT-3´

vascular cell adhesion molecule 1 (VCAM1)

forward, 5’- CCGGCTGGAGATATTAC-3’

reverse, 5’-TGTATCTCTGGGGGCAACAT-3’

intercellular adhesion molecule 1 (ICAM1)

forward 5´- GCAGACAGTGACCATCTACAGC-3´

reverse, 5´-CCATACAGGACACGAAGCTC-3´

IκBα

forward 5´-CAGCAGCTCACCGAGGAC-3´

reverse 5´-ACAGCCAAGTGGAGTGGAGT-3´

metalloproteinase 10 (MMP10)

forward 5´-AGAAGGTAAGGGCAGTGAGAATG-3´

reverse 3´-TTTCTAGGTATTGCTGGGCAAGAT-3´
