# Supplementary material for: A Symphytum officinale Root Extract Exerts Anti-inflammatory Properties by Affecting Two Distinct Steps of NF-κB Signaling
Source: Front Pharmacol. 2019 Apr 26;10:289. doi: 10.3389/fphar.2019.00289 (PMC6498879; doi:10.3389/fphar.2019.00289)
Supplement: Supplementary file 5 [file Data_Sheet_1.PDF]

Supplementary Material - Preparation of comfrey root extract, characterization and quantification of the main compounds

Extraction procedure - 2 Kg of collected roots were washed with cold water, thinly cut and then extracted with a solution of ethanol/water 60% (v/v) (1L) and stored in dark at room temperature (20 °C). After 12 days, the extract was filtered through a coarse-filter. After filtration, a cation-exchange resin was added to the extract to remove pyrrolizidine alkaloids (PA) and 20% aqueous ethanol was added to obtain a liquid extract with a drug to extract ratio (DER) of 2:1. To remove the mucilage, the comfrey root liquid extract 2:1 was treated as successively described. Ethanol was removed from 100 g of liquid extract by rotavapor. Next, the aqueous phase was partitioned with ethyl acetate (1:1) and the organic phase was dried yielding 440 mg.
For HPLC-UV a solution of 100 mg/mL in methanol was prepared (injection volume 20 μL), while for LC-MS analysis a solution of 0.1mg/mL in methanol (LC-MS grade) was used (injection volume 2 μL).

Materials - Water, acetonitrile, methanol and formic acid of LC-MS grade were obtained from VWR (Milan, Italy). Allantoin standard was purchased from Sigma-Aldrich. For NMR analysis methanol-d_4 (99.95%, Sigma-Aldrich) was used.

HPLC Analysis - HPLC analysis was performed using an Agilent 1260 Infinity system (Agilent Technologies, Palo Alto, CA, USA), equipped with a binary pump (G-1312C), and an UV detector (G-1314B). The analysis was carried out at room temperature without thermostating using a Phenomenex C18 Synergi-Hydro-RP (250 mm × 10 mm, 10 μm) column. The mobile phase consisted of solvent A (H_2O + 0.1 % formic acid) and solvent B (CH_3CN + 0.1% formic acid). A flow rate of 2 mL/min was used. The linear gradient program started from 0 to 5% B in 5 min; from 5 to 20% B in 15 min; from 20 to 100% B in 20 min. The detection wavelength was set at 280 nm.

NMR analysis - NMR experiments were acquired in methanol-d_4 (99.95%, Sigma-Aldrich) on a Bruker DRX-600 spectrometer (Bruker BioSpin GmBH, Rheinstetten, Germany) equipped with a Bruker 5 mm TCI CryoProbe at 300 K. Data processing was carried out with Topspin 3.2 software.

Quantitative analysis - Quantification of compounds was performed using a Shimadzu Nexera LC system in line with a Sciex 6500 QTrap MS equipped with a kinetex EVO C18 column (Phenomenex) (100 x 2.1 mm i.d., 5 μm). Mobile phases consisted of water acidified with 0.1%

formic acid (solvent A) and acetonitrile acidified with 0.1% formic acid (solvent B). Compounds were chromatographically separated using the following gradient elution profile at a flow rate of 0.2 ml/min: 0-25 min a linear gradient 5%-95% B and then back to 5%B for 3 min. The 6500 QTrap was configured for IonSpray operation and the compounds were detected using multiple reaction monitoring (MRM) in negative ion mode. Supplementary Figure 1B gives the transitions of all the analytes reported here as well as their collision energies (CE). Values of additional QTrap parameters are as follows: curtain gas (CUR) = 35; collision gas (CAD) = medium; IonSpray voltage (IS) = -4500; temperature (TEM) = 350; ion source gas 1 (GS1) = 25; ion source gas 2 (GS2) = 25; Declustering Potential (DP), Entrance Potential (EP), Collision Energy (CE) values were determined by infusing selected standards into the 6500 QTrap and adjusting the values to obtain the maximal response. Data acquisition and processing were performed using Analyst software 1.6.2 (ABSciex, Foster City, CA, USA). The measurements were done in triplicate.

1 mg of each standard was accurately weighted and dissolved into a 1 mL of methanol:water (50:50 (v/v)) to prepare a stock solution at 1 mg/mL. The stock solution was diluted with methanol in order to obtain work solutions containing 0.01, 0.1, 0.5, 1, 5, 10 μg/mL of standards. The calibration curves for each compound were calculated by regression analysis, by plotting the peak area obtained after standards injection (3 replicates at each concentration) against the known standard concentrations.

Method validation - The LC-ESI-QTrap-MS/MS method was validated according to the European Medicines Agency guidelines (EMEA Quality guidelines ICH Q2) relating to the validation of analytical methods in particular precision, specificity, linearity, limit of quantification (LOQ) and limit of detection (LOD). Precision was evaluated at five concentration levels for each compound through triplicate intra-day assays and inter-day assays over 3 days. Specificity was defined as the non-interference by other analytes detected in the region of interest. Linearity was evaluated by correlation values of calibration curves.
